# Feasibility and Safety of Home-Based Preoperative Management of Selected Lower Extremity Trauma

**DOI:** 10.3390/diagnostics16030432

**Published:** 2026-02-01

**Authors:** Eyal Yaacobi, Tal Shachar, Omer Marom, David Segal, Dan Perl, Nissim Ohana

**Affiliations:** 1 Orthopedic Department, Meir Medical Center, 59 Tchernichovsky Street, Kfar Saba 4428164, Israel; 2 Gray Faculty of Health Sciences, Tel Aviv University, Tel Aviv 6997801, Israel

**Keywords:** home-based preoperative management, lower extremity trauma, elective surgery, postoperative outcomes, healthcare optimization, Emergency Department

## Abstract

**Background/Objectives**: Efficient allocation of hospital resources is crucial in managing lower extremity trauma. Selected patients with stable injuries may not require inpatient hospitalization while awaiting surgical fixation. This study describes the feasibility and safety of a structured **H**ome-based **P**reoperative **M**anagement (HPM) pathway for such patients. **Methods**: We conducted a retrospective, single-center observational study of 187 adult patients with isolated lower extremity fractures managed with HPM between 2019 and 2022. All patients were discharged home from the Emergency Department with standardized instructions, immobilization, anticoagulation, and planned follow-up. No comparator group was included. **Results**: Of 187 patients (mean age 49.7 y), 23 patients (12.3%) returned to the Emergency Department during the preoperative waiting period. The mean time from Emergency Department presentation to surgery was 8.5 days. Overall, 164 patients (87.7%) completed the preoperative waiting period at home without requiring an additional Emergency Department visit. Within one year after surgery, 51 patients (27.3%) presented to the Emergency Department; 29 of these visits (56.9%) were considered surgery-related. Patients who returned to the Emergency Department before surgery had a higher likelihood of postoperative Emergency Department visits within one year compared with those who did not (69.6% versus 21.3%, *p* < 0.001). Time to surgery was not associated with postoperative Emergency Department visits (*p* = 0.763). **Conclusions**: In this retrospective cohort, Home-Based Preoperative Management was feasible and appeared safe for carefully selected patients with lower extremity trauma. Most patients were able to await surgery at home without unplanned Emergency Department visits. Given the absence of a comparator group, no conclusions regarding comparative effectiveness or superiority over inpatient management can be drawn.

## 1. Introduction

Lower extremity trauma (LET) is one of the most prevalent injury patterns encountered in Emergency Departments (EDs). Surgical interventions for these injuries carry inherent risks, including infection, complications in wound healing, delayed union or non-union, anesthetic challenges, and potential damage to nerves, tendons, or blood vessels [1,2,3]. Studies show that the timing of surgical intervention influences postoperative complications and overall outcomes [4,5]. However, the debate between immediate and delayed surgery for LET remains unresolved. In many cases, surgical timing is influenced not only by injury-related factors but also by system-level constraints. Due to their limited mobility, patients with LET pose challenges for the care team in ensuring safe discharge while maintaining adequate nursing care at home. In public healthcare systems, operating room availability often determines surgical timing as much as fracture characteristics [6,7].

During the COVID-19 pandemic, many centers expanded the existing practice of discharging selected orthopedic trauma patients to await surgery at home to preserve inpatient capacity and limit exposure [8,9]. These adaptations highlighted the potential role of structured, non-inpatient pathways for selected trauma patients, while also underscoring the need for careful patient selection, clear instructions, and planned follow-up. The evolution of perioperative care, particularly with the increased use of regional anesthesia and minimally invasive surgical techniques, has significantly reduced the need for prolonged postoperative hospital stays [8,9]. Nevertheless, these advances primarily affect postoperative management and do not directly address the preoperative waiting period for acute fractures.

Home-based Preoperative Management (HPM) is a structured pathway that keeps selected patients at home before surgery with standardized instructions, analgesia, anticoagulation, and scheduled follow-up. The novelty of this approach lies primarily in its organizational and logistical design rather than in any modification of surgical technique or clinical treatment. The existing literature focuses mainly on elective pathways and inpatient management rather than home-based preoperative care for acute fractures, leaving limited evidence regarding feasibility, safety and implementation in the trauma setting [10,11].

Accordingly, the aim of this study was to describe the feasibility and safety of a structured HPM pathway for carefully selected patients with lower extremity trauma awaiting surgical fixation. Specifically, we examined patterns of unplanned Emergency Department revisits during the preoperative waiting period and within one year after surgery as pragmatic indicators of pathway safety. Given the retrospective, single-arm design, this study was not intended to assess comparative effectiveness or superiority over inpatient preoperative management.

## 2. Material and Methods

### 2.1. Study Design and Setting

Retrospective observational single-center cohort study conducted between 2019 and 2022.

### 2.2. Ethical Considerations and Consent to Participate

This study was approved by the Institutional Review Board (IRB) of our hospital (Reference number: MMC-0107-22). This study was conducted in accordance with the principles outlined in the Declaration of Helsinki. As this is a retrospective study, the IRB waived the requirement for informed consent. However, every effort was made to protect patient confidentiality, and all data were de-identified before analysis.

### 2.3. Patients

Inclusion criteria consisted of adult patients (≥18 years) presenting to the Emergency Department (ED) with isolated, closed lower extremity fractures- such as ankle fractures, tibial shaft fractures, or tarsal/metatarsal fractures- that were clinically and radiographically deemed stable for delayed fixation. Eligible patients were medically stable, able to ambulate non-weight-bearing with assistance, and had sufficient social support to manage home care.

Exclusion criteria included open fractures, polytrauma (Injury Severity Score > 15), fractures with neurovascular compromise, pathological fractures, or any injury deemed by the attending orthopedic surgeon to require urgent or immediate surgical intervention. All femoral fractures (neck, proximal, shaft, or distal), whether open or closed, were categorically excluded.

Patients were carefully selected for temporary home care until surgery based on three key principles:

1. The nature of the pathology permitted a waiting period of 7–10 days before surgery.
2. The patient possessed the capability to provide self-care or had a suitable support system in place.
3. The pathology, protected by a cast, allowed for limited mobility.

These criteria were based on institutional orthopedic protocols and expert consensus within the department. The decision to discharge a patient for home-based preoperative management was made by the attending orthopedic surgeon following clinical evaluation and radiographic confirmation. All attending orthopedic surgeons used the same departmental discharge criteria and the same written HPM protocol throughout the study period.

All patients were fitted with the appropriate immobilization device (e.g., short leg cast, long leg cast, or splint) prior to discharge from the Emergency Department, according to the specific fracture type and stability requirements outlined in Table 1. Each patient was provided with a printed copy of the discharge instructions at the time of ED discharge. These instructions (summarized in Table 1) included detailed information on mobility restrictions, cast care, anticoagulation schedules, pain management regimens, physiotherapy guidance, and warning signs requiring return to the ED. Patients were also instructed on the use of mobility aids (e.g., crutches or wheelchairs) and given contact information for follow-up support. For patients prescribed subcutaneous anticoagulant therapy, the ED nursing staff provided hands-on instruction on proper injection technique. To preserve the possibility of same-day surgical scheduling, patients were specifically instructed to administer the injection in the late afternoon (between 16:00 and 17:00), ensuring at least 12 h before anesthesia if called for surgery the next morning.

**Figure 1:**
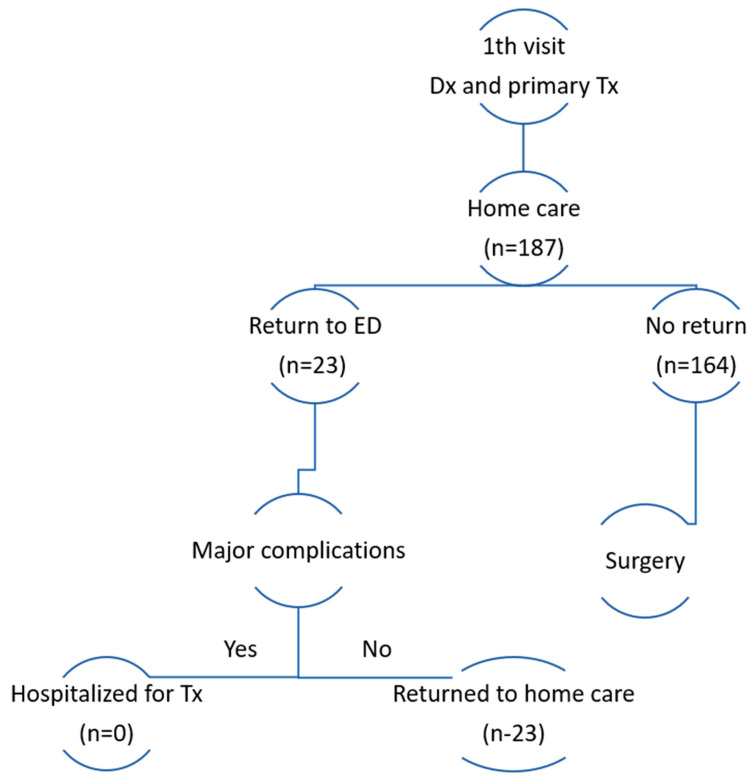
Flowchart of patient selection and outcomes in the Home-based Preoperative Management (HPM) cohort.

The administration of these instructions was overseen by healthcare providers specializing in orthopedics. A vital element of this patient-centered approach was the establishment of continuous communication between the patients and the hospital until the surgical procedure was scheduled. This ensured that patients remained well-informed and supported throughout the preoperative phase (Figure 1).

During their preoperative care period in the comfort of their homes, patients benefited from a continuous stream of medical advice and assistance provided by a multidisciplinary team of healthcare professionals. We used a simple multidisciplinary HPM team, including an orthopedic surgeon, an ED nurse educator, a community nurse, a physiotherapist, an occupational therapist, a social worker, and an OR scheduling coordinator. Their tasks were to

- Give standard discharge teaching and written instructions;
- Confirm the anticoagulation schedule and the analgesia plan;
- Coach non-weight-bearing and safe use of crutches or wheelchair;
- Book the surgery date and update the patient on any changes;
- Make a follow-up call within 24–48 h, with additional calls as needed;
- Triage red flags and arrange return to the ED when needed.

### 2.4. Data Collection

We identified eligible patients by querying the hospital electronic health record for 2019–2022 ED encounters using ICD-9-CM fracture diagnosis codes for ankle, tibial shaft, fibula, tarsal, metatarsal, talus, calcaneus, and patella. We then applied the inclusion and exclusion criteria and deduplicated multiple visits to retain the index presentation. Patient records were retrieved from the hospital’s electronic database using ICD-9 diagnosis codes. The inclusion and exclusion criteria are detailed above.

To maintain consistency across years, we used a predefined data dictionary, trained abstractors on a standard operating procedure, and harmonized variable names and coding across 2019–2022. We performed periodic cross-checks, including random audits of 5% of records per year.

Postoperative outcomes and ED revisits were obtained from the institutional electronic record. When an outside ED visit was suspected or documentation was incomplete, the research team verified details by telephone with the patient or the outside facility.

The data are divided into three distinct sections below.

#### 2.4.1. Section A: Demographic Profile

This section encompasses demographic data, which includes age, gender, and weight, to provide a comprehensive understanding of the patient population.

#### 2.4.2. Section B: Clinical Overview

This section delves into the clinical aspects, covering pertinent information such as prior Emergency Department (ED) visits, the circumstances leading to the ED visit, the diagnosis made during the ED visit, any additional ED visits before surgery, and the rationale behind these visits.

#### 2.4.3. Section C: Surgical Details

This section focuses on surgical aspects, including key information such as the date of the surgical procedure, the duration between ED admission and surgery, the length of the operation, the ASA score (American Society of Anesthesiologists score), the number of postoperative ED visits, and the underlying reasons for these visits. The primary outcome of this study was the rate of unplanned Emergency Department (ED) (ED) visits during the home-based preoperative waiting period. These Emergency Department revisits were used as pragmatic indicators of pathway safety rather than as direct measures of surgical or medical complications. Secondary outcomes included the incidence of postoperative ED visits within one year, reasons for ED visits, and the relationship between time to surgery and complication rates.

Primary outcome: Unplanned ED visit during the home-based preoperative waiting period.

Secondary outcomes: Postoperative ED visit within 365 days, the count and reasons for postoperative ED visits, and the association between time to surgery and complications.

Definition of “surgery-related” ED visit: An ED presentation attributable to the index injury or its surgery, including wound drainage, bleeding, dehiscence, or infection; uncontrolled pain related to the fracture or cast; cast-related problems; new or worsening neurovascular symptoms; suspected deep-vein thrombosis or pulmonary embolism; loss of reduction; or concern for compartment syndrome. Visits for unrelated conditions (e.g., respiratory illness, unrelated trauma) were not classified as surgery-related.

### 2.5. Data Management and Statistical Analysis

Descriptive statistics were used to present the raw data. Continuous variables were summarized as mean (standard deviation) or median (interquartile range) as appropriate; categorical variables as counts and percentages. Categorical variables were compared using a Chi-square test or the Fisher exact test when needed, and continuous variables were compared with Student’s t test, or the Mann–Whitney U test if normality could not be assured. Normality was assessed with the Shapiro–Wilk test and inspection of Q–Q plots. We report effect estimates with 95% confidence intervals for principal outcomes. We planned multivariable logistic regression models for preoperative unplanned ED return and for postoperative ED visit within one year with age sex fracture type ASA class and time to surgery as covariates. Because the number of outcome events was small, we did not fit these models to avoid overfitting. We report unadjusted effect estimates with 95 percent confidence intervals. Results are presented as unadjusted effect estimates with 95% confidence intervals. All analyses were descriptive or unadjusted; no adjusted associations were tested or inferred.

Missing data handling: we quantified missingness for each variable. Analyses used complete cases; if missingness exceeded 10% for a key variable, we performed a sensitivity analysis using multiple imputations with chained equations. The number of missing observations per variable is reported in the Results section. No formal sample-size calculation was performed; all eligible cases from 2019 to 2022 were included.

A *p* value of 0.05 was set for statistical significance. All tests were two-sided. IBM SPSS statistics 28.0 (Armonk, NY, USA) was used for statistical analysis.

## 3. Results

The study cohort comprised 187 patients, with a mean age of 49.65 y (standard deviation = 18.7 y). Among them, 110 individuals (59%) were male and 77 (41%) were female. Patient presentations were distributed across the study period, with the highest proportion in 2022 (29.5%), followed by 2019 (26.2%), 2020 (22.4%) and 2021 (21.9%). Baseline and perioperative characteristics of the study cohort, stratified by preoperative Emergency Department return status, are summarized in Table 2.

Emergency Department utilization during the preoperative waiting period and within one year after surgery is summarized in Table 3. During the preoperative waiting period, 23 patients, (12.3%; 95% confidence interval [CI], 8.3–17.8) returned to the Emergency Department. The mean time from Emergency Department presentation to surgery was 8.5 days. Within one year after surgery, 51 patients (27.3; 95% CI, 21.4–34.1) presented to the Emergency Department. Of the postoperative visits, 29 (56.9%; 95% CI, 43.3–69.5) were classified as surgery-related.

Patients who returned to the Emergency Department during the preoperative waiting period, had a higher proportion of postoperative Emergency Department visits within a year compared with those who did not (69.6% versus 21.3%). This corresponded to an absolute difference of 48.2 percentage points (95% CI, 28.4–68.0). The mean time to surgery was longer among patients who returned preoperatively by 3.0 days (95% CI, −2.0–8.0), although this difference was not statistically significant.

Missing data were minimal and are summarized in Table 2. No missing values were observed for the primary outcomes. Table 2 presents baseline and perioperative characteristics stratified by preoperative Emergency Department return status, with counts and percentages for categorical variables and means with standard deviations for continuous variables.

Among patients who returned to the Emergency Department before surgery, cast-related complaints were the most frequent reason for presentation, accounting for more than half of preoperative returns. Reasons for preoperative Emergency Department return are detailed in Table 3 and included the need for additional imaging, soft tissue infection, limb pain, and unrelated events such as traffic accidents or fever.

Patients who revisited the Emergency Department during the preoperative period also demonstrated a higher frequency of Emergency Department visits during the first postoperative year. However, the proportion of surgery-related postoperative visits was similar between patients who returned preoperatively and those who did not (57.1% vs. 56.3%, *p* = 0.238). No statistically significant differences were observed between groups with respect to fracture type, age, weight, ASA class, operative duration, or hospital length of stay (all *p* > 0.05). Time to surgery was not associated with postoperative Emergency Department visits (*p* = 0.763). No adjusted analyses were performed because of low event counts.

**Figure 2:**
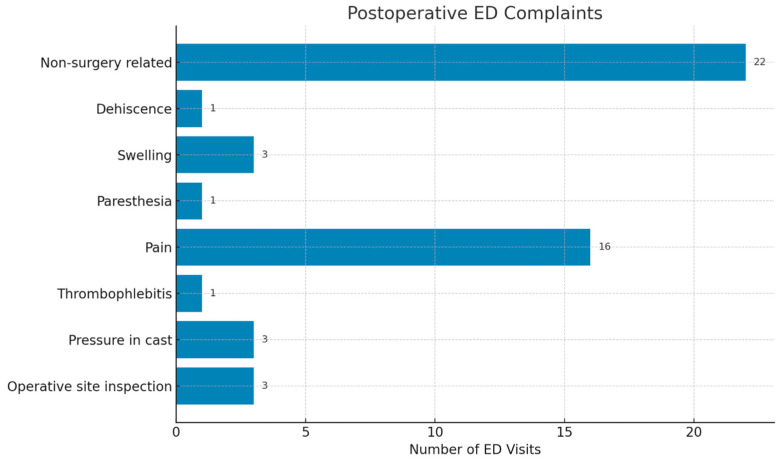
Postoperative Emergency Department visits by reason with counts and percentages.

In total, 51 postoperative ED visits were recorded. Non-surgery-related causes were the most common reasons for postoperative presentation (22 of 51 visits; 44%; 95% CI, 31.2–57.7). Followed by pain in the operated limb (16 of 51 visits; 32%; 95% CI, 20.8–45.8) (Figure 2). Non-surgery-related visits most frequently involved generalized limb swelling, medication-related side effects, unrelated minor injuries, non-specific symptoms (such as fatigue or dizziness), or administrative concerns. All postoperative ED visits were managed on an outpatient basis, and no hospital admissions were required.

## 4. Discussion

This study introduces a structured, organizational approach to Home-based Preoperative Management (HPM) for selected patients with non-life-threatening lower extremity trauma (LET), characterized by clear discharge criteria and planned follow-up. In the absence of a comparison group, the findings of this study should be interpreted as observations of feasibility and safety only. They do not allow for conclusions regarding reductions in complications, Emergency Department (ED) utilization, or superiority over inpatient preoperative care. We did not adjust for confounders because outcome counts were low and a multivariable model would be unstable. Accordingly, causal inferences cannot be made. A prospective study with an appropriate control group would be required to assess comparative effectiveness.

Unlike conventional elective surgeries, which are scheduled well in advance, this approach allows patients to remain at home during the preoperative waiting period with predefined safety checks and return pathways. Orthopedic trauma often requires surgical intervention but does not always necessitate immediate operative treatment, making this strategy a pragmatic option for selected stable injuries. The timing of surgeries under this strategy depends on multiple factors, including the patient’s medical condition, injury characteristics, staff availability, and, in public healthcare systems, competition for operating room resources [12].

The home-based preoperative approach represents a shift in preoperative care organization rather than a change in surgical management. At the system level, potential advantages may include fewer inpatient admission days, reduced ED boarding, and greater flexibility in operating room scheduling. These system-level effects were not directly measured in this study and should be regarded as hypotheses rather than demonstrated outcomes. We suggest that future evaluations track metrics such as bed days saved per 100 HPM patients, unplanned ED use, same-day surgery rates, and staff time per case. Recent reviews report that telemedicine pathways in orthopaedics and trauma support preoperative optimization, diagnosis, conservative care and postoperative follow-up, which aligns with the conceptual framework of HPM [13,14].

Our results indicate that many patients were able to await surgery at home without requiring interim ED visits, supporting the feasibility of this approach in a carefully selected population. By allowing patients to remain at home during the preoperative period, healthcare systems may be better positioned to prioritize more urgent cases and allocate resources more efficiently. This consideration is particularly relevant in resource-constrained environments, where operating room capacity is limited. While our cohort did not include a formal cost analysis, we recommend future service evaluations that compare pre- and post-implementation bed days, same-day discharge rates, and unplanned care utilization. We avoid repeating detailed numerical results here and refer the reader to the Results section. System-level confirmation of resource effects will require prospective comparisons with pre-protocol practice and standardized outcome measures [14,15].

The apparent success of this approach appears to depend largely on careful patient selection. Key elements included stable fracture patterns, reliable social support, and the ability to comply with non-weight-bearing and clear escalation rules. HPM differs from routine outpatient discharge by standardizing discharge instructions, anticoagulation timing, and proactive contact, rather than relying on ad hoc counseling alone. While advances such as regional anesthesia and minimally invasive techniques may contribute to reduced postoperative admissions, these relate to postoperative care and were not the focus of our study. Our findings specifically support the feasibility and safety of managing selected patients at home during the preoperative period [10,11]. Where mechanisms are discussed, they are presented as hypotheses rather than observed effects. In this context, HPM may provide a viable framework for addressing the long-standing debate regarding the optimal timing of surgery for selected LET patients [4,5]. The lack of association between wait time and postoperative ED visits suggests that, for stable fractures, safety may depend more on patient selection and education than on a strict time-to-surgery threshold.

Several challenges remain. Operational risks include inadequate pain control, cast-related problems, and inconsistent adherence to non-weight-bearing and anticoagulation instructions. Logistical issues, such as ensuring patients have access to appropriate home care and monitoring, must also be addressed. To strengthen safety, we suggest simple telehealth support, including daily symptom prompts, brief photo checks of casts or toes, a scheduled virtual review before surgery, and medication reminders with clear rules that trigger an in-person assessment. Recent reports in orthopaedics and trauma support the feasibility of these tools. Future research should focus on refining patient selection criteria and developing guidelines to optimize the implementation of HPM in diverse healthcare settings. In response to the reduced hospitalization rates during the COVID-19 pandemic [16], our department adopted HPM proactively and now considers it a permanent practice rather than a temporary pandemic measure.

In this cohort, most patients were able to await surgery without requiring ED reassessment; among those who did return, cast-related issues were the most common reason (Table 3). During the post-operative period, pain was the primary reason for ED presentation. All ED visits were managed on an outpatient basis, but this finding should be interpreted cautiously given the sample size. Female patients had higher ED revisit rates; possible explanations are presented as hypotheses only. Possible contributing factors include differences in pain reporting, caregiving responsibilities, or sex-related differences in soft tissue response, as well as cast fit or comfort. These hypotheses warrant further investigation using patient-reported outcomes, structured pain assessments, and measures of social support in larger cohorts. Most postoperative ED visits were for non-surgery-related causes. No adjusted analyses were performed because of the low number of events; therefore, independent associations remain uncertain. We avoid repeating numeric estimates and refer the reader to the results section. As described in the Methods, anticoagulation was managed according to a standardized protocol; no venous thromboembolism events were observed during follow-up in this cohort.

To the best of our knowledge, no previous studies have specifically examined a semi-elective, structured home-based preoperative approach for lower extremity trauma patients awaiting surgery. The present study provides observational feasibility and safety data in carefully selected patients. Importantly, the HPM protocol was intentionally designed to rely on standard clinical roles and routine care processes, allowing replication in other healthcare settings with local organizational adaptation rather than dependence on unique institutional resources. Longer preoperative waiting times were not associated with increased postoperative ED utilization in this cohort. These findings should not be interpreted as evidence of improved outcomes or reduced complications. Future studies should prospectively compare HPM with inpatient preoperative care and evaluate telehealth-supported monitoring using outcomes such as patient experience, cost and unplanned care.

## 5. Limitations

This study is subject to selection bias, as patients chosen for HPM were clinically stable and had reliable support at home; therefore, the findings may not be generalizable to the broader lower extremity trauma population.

This was a single-center, retrospective study without a comparison group; accordingly, causal inferences cannot be made, and superiority over inpatient preoperative care cannot be claimed.

Event counts were relatively low; therefore, multivariable analyses were not performed, and independent associations remain uncertain.

Long-term outcomes, such as functional recovery, pain, or patient satisfaction, were not assessed.

Follow-up relied primarily on institutional medical records, supplemented by telephone contact when needed; Emergency Department visits at outside institutions may therefore have been missed. In addition, bed days saved and direct costs were not measured, precluding assessment of the system-level economic impact of this approach. Accordingly, the findings should not be interpreted as evidence of equivalence or non-inferiority compared with inpatient preoperative management.

## 6. Conclusions

Home-based preoperative management (HPM) was feasible and appeared safe in carefully selected patients with lower extremity trauma. Most patients were able to await surgery at home without requiring unplanned Emergency Department visits during the preoperative period. Given the observational, single-arm design, no conclusions can be drawn regarding reductions in complications or comparative effectiveness relative to inpatient preoperative care. This model may offer a pragmatic organizational option for selected patients in public healthcare systems facing resource constraints. Future prospective studies with appropriate comparator groups are required to evaluate comparative effectiveness, resource utilization, and patient-centered outcomes.

## Figures and Tables

**Table 1:** Fracture-specific guidance on immobilization, mobility, medication, and red-flag symptoms.

	Fracture Type						
	Bimalleolar/Tri Malleolar	Malleolus (Lateral/Medial)	Patella	Fibula or Tibia	Calcaneus	Talus	Tarsal or Metatarsal
Mobility	NWB	NWB	FWB	NWB	NWB	NWB	NWB
**Cast**	Short leg cast	Short leg cast	Tutor cast	Long leg cast OR Posterior long ankle splint	Short leg cast	Short leg cast	Short leg cast
**Physio**	Maintain knee movement. Static quadriceps muscle contraction and toes movement. Instruction for use of crutches/wheelchair	Maintain knee movement. Static quadriceps muscle contraction and toes movement. Instruction for use of crutches/wheelchair	Full activity	Instruction for use of crutches/wheelchair	Maintain knee movement. Static quadriceps muscle contraction and toes movement. Instruction for use of crutches/wheelchair	Maintain knee movement. Static quadriceps muscle contraction and toes movement. Instruction for use of crutches/wheelchair	Maintain knee movement. Static quadriceps muscle contraction and toes movement. Instruction for use of crutches/wheelchair
Anticoagulants *	Enoxaparin 40 mg 1 × 1/d						
**Pain according to VAS** VAS 4–6/10 OR VAS 6–7 VAS 7–10/10 OR VAS 8–10	Paracetamol 1 g 1 × 4/d Paracetamol 1 g 1 × 4/d + Tramadol 100 mg 1 × 1/d OR Tramadol HCL 37.5 mg + paracetamol 325 mg 1 × 3/d OR Oxycodone + Naloxone 10 mg 1 × 2/d						
**Symptoms requiring ED re-visit**	Out of proportion pain Limb numbness Fever Tightness around cast Dyspnea						
**Decision for surgery**	During ER consult or Within 24–48 h (exluding weekend/holidays)						
**Patient update**	Immediate after decision making, date for surgery within few days						

Abbreviations: NWB: Non-weight-bearing; FWB: Full weight bearing; VAS: visual analog scale; ED: Emergency Department. * Corrected for GFR and according to patient weight: <50 kg: 20 mg 1 × 1/d, 50–80: 40 mg 1 × 1/d, >80 kg: 60 mg 1 × 1/d.

**Table 2:** Baseline and perioperative characteristics of the study cohort stratified by preoperative Emergency Department return.

Variable	Did Not Return to ED Before Surgery (*n* = 164)	Returned to ED Before Surgery (*n* = 23)	Total (*n* = 187)	*p* Value	Missing, *n*
Age, years (mean ± SD)	49.65 ± 18.70	50.70 ± 20.55	49.78 ± 18.89	0.804	0
Male sex, *n*/*N* (%)	101/164 (61.6)	9/23 (39.1)	110/187 (58.8)	0.035	0
Weight, kg (mean ± SD)	64.39 ± 32.78	53.09 ± 37.35	63.00 ± 33.48	0.130	NA
**Fracture site (AO/OTA), *n/N* (%)**				0.470	NA
• Foot	15/164 (9.1)	0/23 (0.0)	15/187 (8.0)	NA	NA
• Ankle	106/164 (64.6)	17/23 (73.9)	123/187 (65.8)	NA	NA
• Tibial shaft	11/164 (6.7)	2/23 (8.7)	13/187 (7.0)	NA	NA
• Tibial plateau	15/164 (9.1)	3/23 (13.0)	18/187 (9.6)	NA	NA
• Patella	17/164 (10.4)	1/23 (4.3)	18/187 (9.6)	NA	NA
**ASA class** (mean ± SD)	1.89 ± 0.65	2.19 ± 0.65	1.98 ± 0.66	0.137	NA
**Duration from ED to OR, days** (mean ± SD)	8.13 ± 7.20	11.16 ± 11.96	8.51 ± 7.96	0.087	0
**Duration of surgery, min** (mean ± SD)	97.1 ± 49.16	95.0 ± 46.09	96.8 ± 48.68	0.844	NA
**Length of stay, days** (mean ± SD)	5.55 ± 8.49	4.06 ± 2.41	5.11 ± 7.24	0.495	NA

ED = Emergency Department; ASA = American Society of Anesthesiologists; OR = operating room. AO/OTA codes: Foot 87.1B, 83–85; Ankle 44-B1, 44-B2, 44-C1; Tibial shaft 42-A2, 42-C2; Tibial plateau 41-B3, 41-C1; Patella 34-C1, 34-C3, 34-A1. Percentages may not total 100% due to rounding.

**Table 3:** Emergency Department utilization and outcomes.

Variable	Did Not Return to ED Before Surgery (*n* = 164)	Returned to ED Before Surgery (*n* = 23)	Total (*n* = 187)	*p* Value	Missing, *n*
**Preoperative ED return, *n/N* (%)**	—	—	23/187 (12.3)	—	0
**Reason for preoperative ED return, *n/N* (% of 23)**					
• Pressure in cast	—	13/23 (56.5)	13/23 (56.5)	—	0
• Cast loosening	—	1/23 (4.3)	1/23 (4.3)	—	0
• Additional imaging	—	3/23 (13.0)	3/23 (13.0)	—	0
• Limb pain	—	1/23 (4.3)	1/23 (4.3)	—	0
• Cellulitis	—	2/23 (8.7)	2/23 (8.7)	—	0
• Traffic accident	—	2/23 (8.7)	2/23 (8.7)	—	0
• Fever of unknown origin	—	1/23 (4.3)	1/23 (4.3)	—	0
**Postoperative ED visit within 365 days, *n/N* (%)**	35/164 (21.3) [95% CI 15.4–28.5]	16/23 (69.6) [95% CI 48.8–84.7]	51/187 (27.3) [95% CI 21.4–34.1]	<0.001	0
**Surgery-related postoperative ED visit, *n/N* (%)**	20/35 (57.1)	9/16 (56.3)	29/51 (56.9) [95% CI 43.3–69.5]	0.238	0

Percentages may not total 100% due to rounding.

## Data Availability

The data presented in this study are available from the corresponding author upon reasonable request. The data are not publicly available due to ethical and privacy restrictions.
